# Emerging trends in cardiovascular diseases: the impact of ferroptosis and cuproptosis on cardiomyocyte death

**DOI:** 10.1007/s11010-025-05340-w

**Published:** 2025-06-23

**Authors:** Peijian Chen, Ping He, Xuejing Rao, Minglu Ding, Jieting Liu, Yanhui Chu, Yang Xiao

**Affiliations:** 1 College of Life Sciences, Mudanjiang Medical University, Mudanjiang, 157011 China; 2 Heilongjiang Key Laboratory of Anti-Fibrosis Biotherapy, Mudanjiang Medical University, Mudanjiang, 157011 China; 3 School of Graduate Studies, Mudanjiang Medical University, Mudanjiang, 157011 China; 4 Department of General Surgery, The Second Affiliated Hospital of Mudanjiang Medical University, Mudanjiang Medical University, No.15 DongXiaoyun Street, Aimin District, Mudanjiang, 157011 China

**Keywords:** Cuproptosis, Ferroptosis, Cardiovascular diseases, Cell death, Molecular mechanism

## Abstract

Cardiovascular diseases (CVDs) comprise a range of conditions affecting the heart and vasculature, encompassing ischemic heart disease (IHD), stroke, heart failure (HF), peripheral and aortic diseases, arrhythmias, and valvulopathy. Notably, the high prevalence rates of CVDs among younger populations warrant concern, emphasizing the importance of prevention and treatment. In recent years, ferroptosis, a novel form of cell death, has attracted significant research interest across various diseases. Similarly, cuproptosis, another cell death mechanism resulting from copper ion accumulation, has also been extensively studied. Cell death plays a crucial role in the development and maintenance of organisms, with both ferroptosis and cuproptosis closely associated with cell metabolism, signaling pathways, and drug resistance. Emerging evidence suggests that ferroptosis and cuproptosis are closely linked to the occurrence and progression of various diseases, including CVDs. The death of myocardial cells is pivotal in the pathophysiology of CVDs, with the roles of ferroptosis and cuproptosis in this process increasingly recognized. This article aims to summarize the molecular mechanisms and interactions of ferroptosis and cuproptosis, as well as their potential as novel targets for CVD treatment.

## Introduction

Cardiovascular diseases (CVDs) represent a significant global health burden, contributing to millions of cases of illness, disability, and premature deaths. According to recent statistics, by 2022, approximately 19.8 million people worldwide are projected to die from CVDs, with a staggering 34% of these deaths occurring before the age of 70 [1]. The development of CVDs often results from the progressive accumulation of adverse factors over time, including hypertension, elevated cholesterol levels, smoking, excessive alcohol consumption, diabetes, sedentary lifestyles, emotional distress, obesity, and genetic predispositions. Current therapeutic approaches for CVDs encompass a range of interventions, such as pharmacological treatments, coronary artery procedures, bypass surgeries, cardiac assistive devices, atrial bypass, and heart transplantation. While these treatments may decelerate the progression of heart failure (HF) to some extent, they exhibit limited ability to fully restore myocardial function. Heart transplantation, although offering a potential remedy for end-stage HF, is severely limited by its high costs and the scarcity of donor organs [2]. Consequently, there is an urgent need for innovative and effective treatments for CVDs, which continue to pose a significant threat to the health and lives of populations worldwide.

Recent studies have identified multiple regulated forms of cell death that play a crucial role in the development of CVDs [3]. Cell death can be triggered by programmed genetic mechanisms, such as apoptosis, necroptosis, and pyroptosis, or by metabolic dysregulation, including ferroptosis and cuproptosis. Iron and copper are essential for various physiological processes, and maintaining precise concentrations of these metals is crucial for optimal cellular growth and proliferation [4]. Dysregulated iron or copper homeostasis can lead to disease, underscoring the importance of understanding the cellular damage caused by imbalances in these metals [5–7]. Identifying and targeting cell death mechanisms in CVDs is crucial for effective treatment. Ferroptosis is an iron-ion dependent form of programmed cell death characterized by cellular damage caused by lipid peroxidation and dysregulated iron metabolism. This form of cell death differs from conventional apoptosis and necrosis in its morphological manifestations of mitochondrial contraction, cristae disruption, and outer membrane rupture. Ferroptosis is now understood to be involved in a variety of pathological processes in cardiovascular diseases, including myocardial ischemia–reperfusion injury, heart failure, cardiomyopathy, and atherosclerosis [8]. Cuproptosis, another emerging mode of cell death, is a form of cell death induced by copper ions that, unlike ferroptosis, involves cellular dysfunction due to an imbalance in copper metabolism. Copper plays a vital role in the cardiovascular system, with excessively high or low levels associated with cardiovascular disease [9]. Both ferroptosis and cuproptosis play essential roles in cardiovascular disease.

Recent studies have suggested a possible mechanistic link between ferroptosis and cuproptosis. Crosstalk between regulated cell death (RCD) pathways, such as apoptosis and necroptosis, has been documented [10]. However, the relationship between cuproptosis and ferroptosis remains relatively understudied. Cell death serves as a defense mechanism against cancer and other proliferative disorders and may be pivotal in the pathogenesis of organs like the heart. This review explored the molecular mechanisms of ferroptosis and cuproptosis, focusing on their interactions and highlighting potential therapeutic strategies for CVD treatment.

## Ferroptosis

Iron, a fundamental element in human biological processes, plays a crucial role in the synthesis of various essential proteases [11, 12]. Maintaining iron metabolic homeostasis is essential for the body’s physiological functions. As an essential component of various metalloproteins, iron plays a key role in biochemical processes, including oxygen transport in tissues, electron transfer during mitochondrial respiration, DNA synthesis and repair, and xenobiotic metabolism [13]. Excessively low or too high iron levels can lead to a variety of diseases [14]. Iron ions typically exist as trivalent ions bound to transferrin, which enter cells through transferrin receptors. Within the cell, these ions are reduced to divalent iron by the metalloreductase six-transmembrane epithelial antigen of prostate 3 (STEAP3), subsequently engaging in various physiological and biochemical processes. Once reduced, divalent iron ions preferentially form complexes that participate in essential biochemical reactions. When these complexes near saturation, any excess divalent iron accumulates, forming a labile iron pool (LIP). Free divalent iron within this pool participates in the Fenton reaction, generating reactive oxygen species (ROS), such as hydroxyl radicals. The accumulation of ROS leads to the peroxidation of membrane lipids, resulting in cellular dysfunction and potentially cell death. This process is a classical pathway in the regulation of cellular ferroptosis [15].

In recent years, ferroptosis has attracted significant interest as a novel form of non-apoptotic cell death. Lipid peroxidation (LPO) clearance failure, redox active iron, and phospholipid oxidation including polyunsaturated fatty acids (PUFA) are regarded as the three basic characteristics of ferroptosis [16, 17]. Morphologically, ferroptosis exhibits reduced mitochondrial volume, increased membrane density, and decreased or absent mitochondrial cristae [15, 18]. However, during ferroptosis, the cell membrane remains intact and the nucleus maintains its normal size, with no chromatin condensation. Biochemically, ferroptosis involves depletion of intracellular glutathione (GSH) and reduced glutathione peroxidase 4 (GPX4) activity. As a result, LPO products metabolism by GPX4 is impaired. Instead, Fe^2^⁺ oxidizes lipids through a Fenton-like reaction, generating significant ROS and promoting ferroptosis [19]. Genetically, ferroptosis is a biologically regulated process influenced by many genes [20].

### Mechanism of ferroptosis

**Fig. 1:**
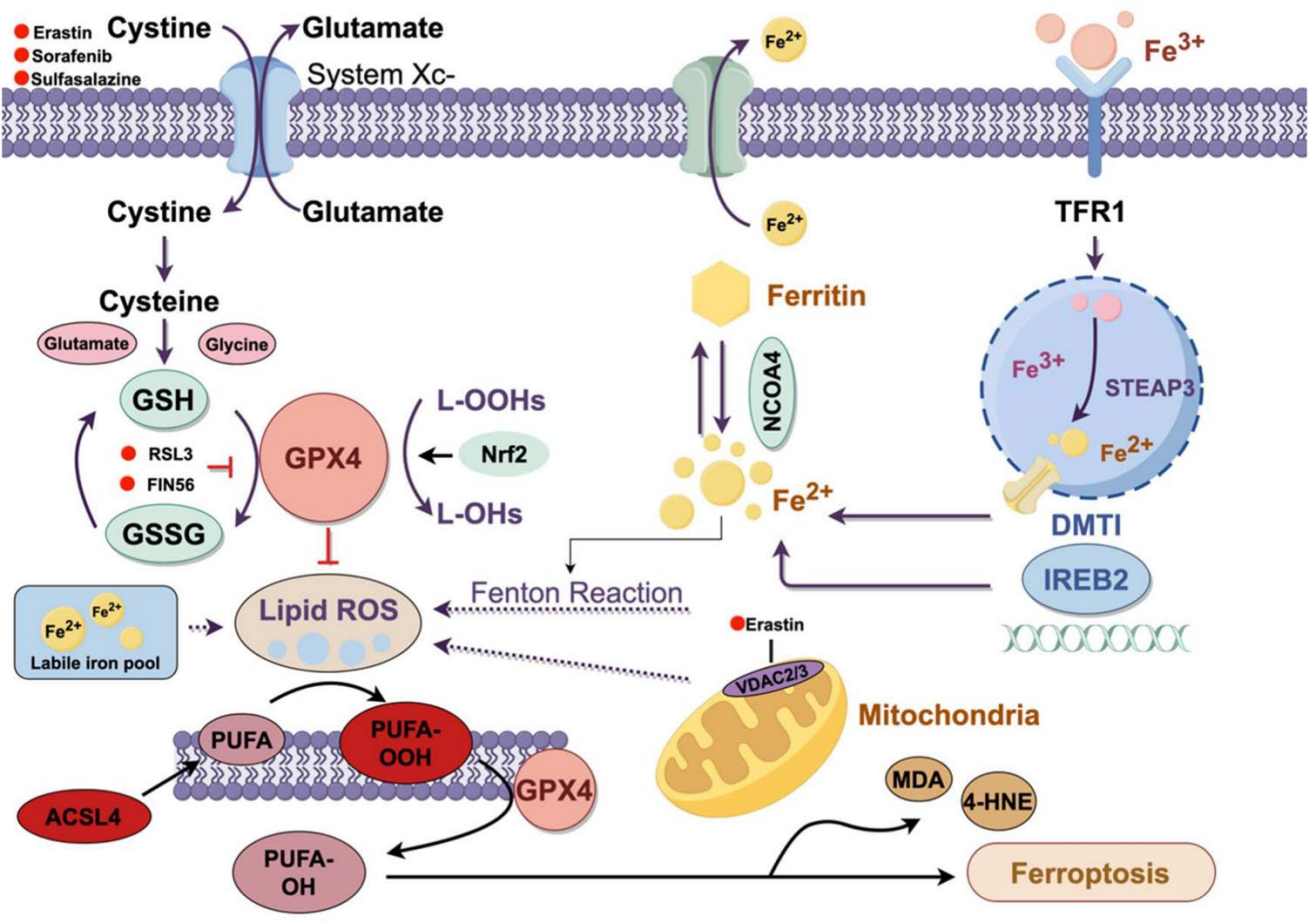
Molecular mechanisms of ferroptosis

Ferroptosis is a form of programmed cell death driven by iron-dependent lipid peroxidation. Its initiation stems from dysregulated intracellular iron metabolism: after binding to transferrin (TF), Fe^3^⁺ enters the cell via transferrin receptor 1 (TFR1)-mediated endocytosis. Within endosomes, Fe^3^⁺ is reduced to Fe^2^⁺ by STEAP3, and subsequently enters the cytoplasmic LIP via divalent metal transporter 1 (DMT1), while excess iron is stored in the ferritin complex consisting of ﻿ferritin heavy chain (FTH) and ﻿ferritin light chain (FTL). Fe^2^⁺ in the LIP is catalyzes by the Fenton reaction to produce ROS and LPO, directly disrupting the cell membrane structure and increasing membrane porosity, ultimately triggering ferroptosis [21]. In this process, polyunsaturated fatty acid coenzyme A (PUFA-CoA) synthesized by lysophosphatidylcholine acyltransferase3 (LPCAT3) and polyunsaturated-fatty-acid–containing phospholipids (PUFA-pls) generated by Acyl-CoA Synthetase Long Chain Family Member 4 (ACSL4) act as key substrates for lipid peroxidation, catalyzing the oxidation of PUFA in membrane phospholipids and generating toxic products, such as 4-hydroxynonenal (4-HNE) and malondialdehyde (MDA), which lead to the impairment of cellular structure and function. System Xc- promotes cellular uptake of cystine, which is subsequently reduced to cysteine by cystine reductase, a precursor of GSH. GSH acts as a key reducing cofactor for GPX4, which can reduce lipid peroxides to the corresponding alcohols and generate oxidized glutathione (GSSG). During ferroptosis, the uptake of extracellular cysteine by the cystine/glutamate reverse transporter protein xCT, consisting of solute carrier family 7 member 11 (SLC7A11) and solute carrier family 3 member 2 (SLC3A2), is reduced, an upstream event in the cascade response to ferroptosis under oxidative stress conditions. Reduced cysteine levels lead to down-regulation of GSH levels and impair the activity of the iron-resistant side-chain phospholipid peroxidase, GPX4. GPX4 acts as an iron-death-specific marker [22] and plays a vital role in attenuating LPO and ferroptosis [23, 24]. Notably, it is the only enzyme capable of converting phospholipid hydroperoxides to phosphatidyl alcohols, which will reduce lipid peroxides to lipocalciferol, convert potentially toxic polyunsaturated L-OOH to nontoxic and less reactive L-OH, and reduce hydrogen peroxide to water during ferroptosis [25].GPX4 inhibits ferroptosis by decreasing ROS production and protects the cells from damage caused by lipid peroxides. ROS interact with polyunsaturated fatty acids in lipid membranes, leading to LPO. It has been shown that treatment of nuclei with homocysteine induces DNA methylation of the nuclear GPX4 gene and enhances sensitivity to ferroptosis [26]. This also suggests that methylation of the GPX4 promoter inhibits GPX expression, leading to accumulation of lipid ROS, which subsequently triggers ferroptosis. In addition, the extra-mitochondrial protein voltage-dependent anion channel2/3(VDAC2/3) sensitizes cells to Erastin-induced ferroptosis. Nuclear factor-erythroid 2 related factor 2(Nrf2) is a key negative regulator of ferroptosis and acts by regulating various downstream genes including SLC7A11, FTH1, and GPX4. Ferritin autophagy is a specific type of selective autophagic degradation of ferritin mediated by nuclear receptor coactivator 4/ferritin heavy chain1(NCOA4/FTH1). Overactivation of ferritin phagocytosis leads to increased iron overload and ferroptosis. Ferroptosis inducers include inhibitors of the xCT/GPX4 axis and FIN56 inhibitors. Conversely, both iron chelators and lipid peroxidation inhibitors both inhibit ferroptosis. (Fig. 1).

## Cuproptosis

In the human body, copper exists as both cuprous ions (Cu^+^) and cupric ions (Cu^2+^). As a vital micronutrient and a key metal in biological systems, copper plays an essential role in the activity of redox-active enzymes. These enzymes are crucial for metabolic reactions, signaling pathways, and various biological functions [27, 28]. However, excess copper can catalyze the production of toxic ROS that are cytotoxic. Accordingly, copper transport and cellular homeostasis are tightly controlled [29]. The human body primarily acquires copper from dietary sources, where exists as Cu^2+^. The intestine plays a pivotal role in regulating copper levels, with enterocytes modulating the absorption of dietary copper. This regulation occurs through the modulation of metallothionein expression and the expression and localization of the high-affinity copper transporter, copper transport protein 1 (CTR1) [30], and the transport ATPase A (ATP7A) [31–33]. After ATP7A-mediated transport to the portal vein, copper is carried to the liver by plasma proteins like albumin and transferrin [34]. The liver serves as a crucial regulator of systemic copper homeostasis, managing copper through sequestration, distribution, and excretion. Copper can be stored within hepatocytes, transported to bile for excretion, or released into the bloodstream for further dissemination. In circulation, copper binds to plasma proteins, such as ceruloplasmin, albumin, and transferrin, facilitating its transport to various organs and tissues [35]. Under conditions of copper overload, the ATPase copper transporting Beta (ATP7B) in hepatocytes facilitates the transport of copper into bile for excretion via the bile ducts [36]. Conversely, dysregulated copper homeostasis elevates intracellular copper concentrations, leading to cellular damage and the phenomenon known as cuproptosis. Copper ions contribute to this process by generating substantial ROS through the Fenton reaction, which induces DNA damage and LPO. Furthermore, copper ions inhibit protease activity by disrupting the ubiquitin–proteasome system and impeding cell proliferation. They also interact with lipid-acylated proteins, such as pyruvate dehydrogenase, which elevates intracellular copper concentrations, leading to cellular damage and aggregation of lipoylated proteins, impairing mitochondrial metabolic functions, thereby promoting cuproptosis. Moreover, copper accumulation is intricately linked to cuproptosis, a process regulated by ferredoxin 1 (FDX1) and lipoic acid synthase (LIAS).

### Mechanism of cuproptosis

The concept of cuproptosis was introduced by Peter Tsvetkov in 2022. Tsvetkov and his colleagues demonstrated that copper induces cell death even when pathways of apoptosis, necrosis, pyroptosis, and ferroptosis are inhibited, indicating that cuproptosis represents a distinct mode of cell death [37]. This process involves the dysregulation of copper metabolism, the accumulation of lipoylated proteins, and the reduction of iron-sulfur (Fe-S) cluster proteins [38, 39]. Copper ionophores, which transport copper into cells, are useful tools for studying copper toxicity. Several lines of evidence suggest that the mechanism of copper ionophore-induced cell death involves the accumulation of intracellular copper rather than the role of the ionophore molecules. Tsvetkov et al. demonstrated that copper ionophores could induce cell death through this mechanism, thereby disrupting the homeostasis of copper in human cells. Cu (II)-Elesclomol was found to be translocated to the mitochondria, where it was reduced to Cu (I), which subsequently induced ROS-dependent apoptosis, given that Cu (II)-Elesclomol treatment does not activate Cu (II)-Elesclomol [40]. The inability of Elesclomol treatment to activate caspase-3, a hallmark of apoptosis, coupled with the observation that inhibiting apoptotic or other known programmed cell death pathways fails to prevent cuproptosis, suggests that cuproptosis is distinct from established forms. Furthermore, mitochondrial aerobic respiration is pivotal in regulating cuproptosis; cells reliant on this process are significantly more sensitive to copper ionophores than those dependent on glycolysis. This heightened sensitivity in cells reliant on mitochondrial respiration suggests a close association with the tricarboxylic acid cycle (TCA cycle). The aggregation of lipoylated proteins is essential for the occurrence of cuproptosis. Protein lipoylation, a highly conserved post-translational modification of lysine, involves the covalent attachment of lipoic acid to lysine residues through amide bonds [41]. Four key enzymes are uniquely associated with the TCA cycle [42]: dihydrolipoctamide branched chain acyltransferase E2, glycine cleavage system protein H, dihydrolipoctamide s-succinyltransferase, and pyruvate dehydrogenase complex acyltransferase dihydrolipoctamide acyltransferase [43]. Copper overload results in the oligomerization of lipoylated proteins, depletion of Fe-S cluster proteins, an increased abundance of heat shock protein 70 (HSP70), and proteotoxic stress, culminating in cuproptosis. Recent studies have identified FDX1 and LIAS as pivotal mediators of cuproptosis. FDX1 is essential for synthesizing steroid hormones, hemoglobin A, and Fe-S proteins. Under FDX1 regulation, LIAS catalyzes the attachment of the thioctanoyl moiety to dihydrolipoamide S-acetyltransferase (DLAT), which is crucial for the mitochondrial pyruvate dehydrogenase complex (PDC) function [44]. Dysregulation of copper homeostasis results in cellular metabolic disorders. Excessively copper ion accumulation mediated by ion carriers or transporter proteins results in the reduction of Cu^2+^ to the more toxic Cu^+^ by FDX1. This reduction inhibits the synthesis of mitochondrial respiration-associated iron-sulfur clusters (Fe-S clusters) and induces a proteotoxic stress response, ultimately leading to cell death. Besides, FDX1 serves as an upstream regulator of protein lipoylation, playing a crucial role in the lipoylation of DLAT [45]. Cu^2+^ can directly bind to and promote the heterodimerization of DLAT, resulting in an increase in insoluble DLAT, which leads to cytotoxic stress and subsequently induces cell death.

**Fig. 2:**
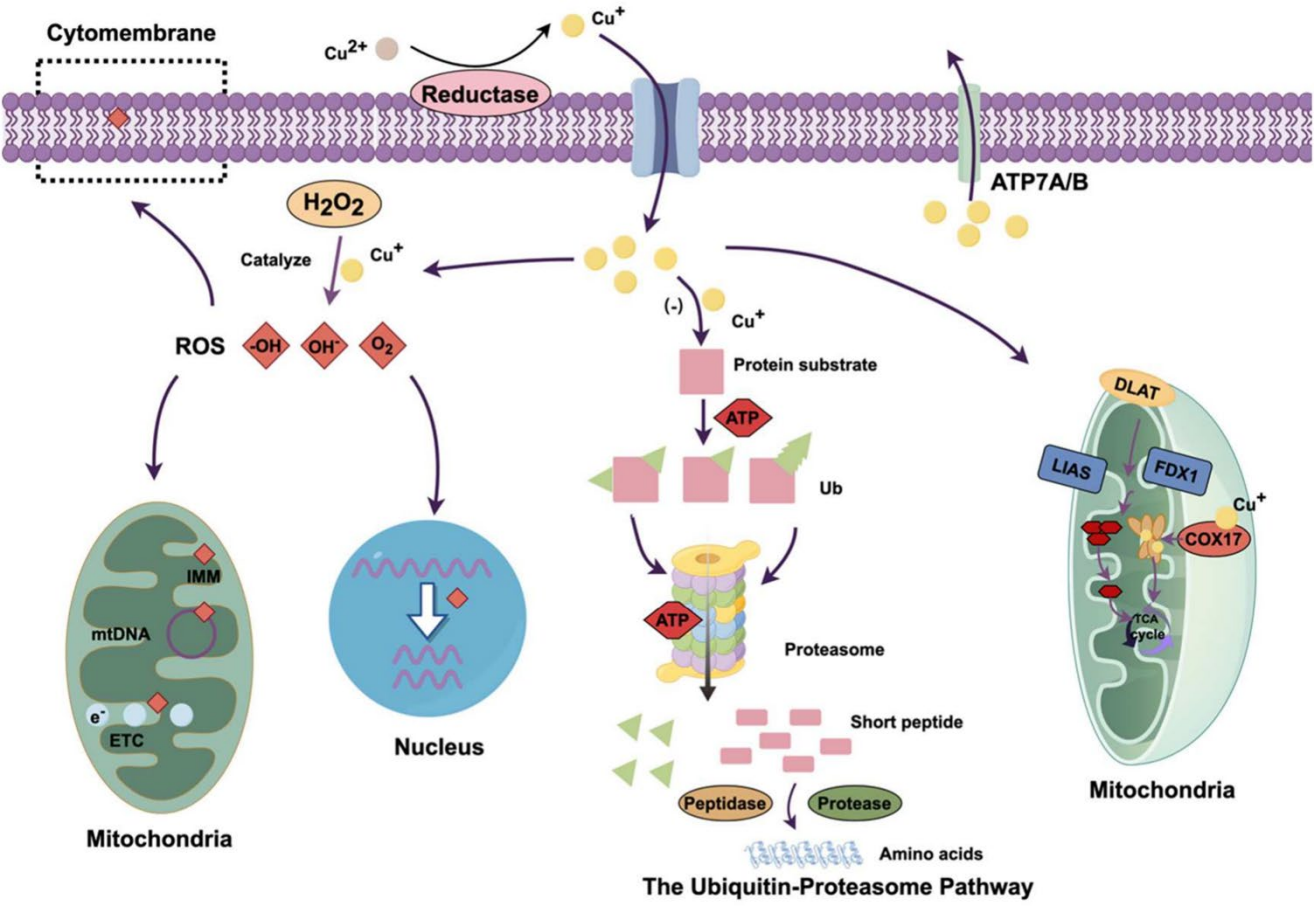
Molecular mechanisms of cuproptosis

Copper ions catalyze the Fenton reaction, generating ROS free radicals. When ROS binding to unsaturated fatty acids on the cell membrane, they induce induces LPO, increasing membrane permeability. This damage extends to the inner mitochondrial membrane, disrupting the mitochondrial electron transport chain (ETC). Furthermore, ROS can compromise the integrity of the DNA double helix, leading to breaks and damage that culminate in cell death. Copper ions also inhibit the ubiquitin–proteasome system, causing a disruption in protein metabolism and affecting cellular function. Moreover, copper ions facilitate the aggregation of lipoylated proteins in the TCA cycle and mediate the depletion of Fe-S clusters, leading to proteotoxic stress and eventual cell death (Fig. 2).

### Triggering and detection of cuproptosis

The past few years have witnessed burgeoning interest in the potential of cuproptosis for the treatment of tumors and other diseases. This process is typically initiated by elevating the intracellular concentration of free copper ions, which involves modulating copper uptake, export, and storage. The cellular influx and efflux of copper ions are regulated by the transport proteins solute carrier family 31 member 1(SLC31A1) and ATP7B [46]. The intracellular concentration of copper ions can be modulated by overexpressing SLC31A1, enhancing copper uptake; knocking down ATP7B to diminish coppe efflux; and employing coppe ionophores such as Elesclomol and Disulfiram [47], which transport extracellular Cu^2+^ directly into the cell; The consumption of endogenous intracellular copper chelating agent GSH through the use of butylthionine sulfoimide (BSO) prevents GSH from chelating free copper ions [48]. Consequently, the surplus Cu^2^⁺, or its more toxic reduced state, is imported into the cell, promoting DLAT oligomerization through binding to thioctylated DLAT. Concurrently, copper ions induce destabilization of Fe-S clusters or inactivation of Npl4-p97, culminating in cell death through a uniquely copper-induced mechanism [49].

The primary morphological characteristics of cell death induced by cuprum include mitochondrial shrinkage, cell membrane rupture, endoplasmic reticulum damage, and chromatin fragmentation. While these features resemble those observed in apoptosis, the underlying mechanisms are distinct [50–52]. In a study by Zhao et al., investigating the correlation between copper ion accumulation and retinal developmental abnormalities and diseases, the morphological characteristics of embryos subjected to copper excess were meticulously measured. TEM analysis uncovered that the endoplasmic reticulum and mitochondrial structures in the retinal cells of copper-treated embryos were significantly compromised. Compared to the control group, these cells exhibited a reduction in the mitochondrial inner membrane and the formation of large vacuoles, while the endoplasmic reticulum displayed a notably loose structure [50]. FDX1 and LIAS have emerged as potential biomarkers for cuproptosis. Research indicates that the copper ionophore Elesclomol triggers copper-induced cytotoxicity in cardiomyocytes, marked by reduced Fe-S cluster proteins and diminished mitochondrial enzyme lipoylation. In 2023, Huo S et al. employed Western blot analysis to identify key changes linked to ES-Cu-induced cuproptosis in muscle cells. The findings revealed a downregulation of various mitochondrial Fe-S cluster proteins, including FDX1, LIAS, Aconitase 2 (ACO2), Electron Transfer Flavoprotein Dehydrogenase (ETFDH), and NADH: ubiquinone oxidoreductase core subunit V1 (NDUFV1), along with reduced lipoylation of DLAT and DLST proteins. Furthermore, protein blot analysis of Fe-S cluster proteins in the cardiac tissue of diabetic (db/db) mice showed a depletion of Fe-S cluster proteins such as FDX1, LIAS, NADH: ubiquinone oxidoreductase core subunit S8 (NDUFS8), and ACO2 in the hearts of db/db mice, accompanied by an increase in HSP70 levels [53]. The functionality of FDX1 is determined by the accumulation of pyruvate and α-ketoglutarate, while avoiding succinate utilization, to maintain the integrity of the TCA cycle, particularly through pyruvate dehydrogenase (PDH) and α-ketoglutarate dehydrogenase, ultimately leading to protein lipoylation. In a study by Tian et al. a nanoadjuvant, CS/MTO-Cu@AMI, was designed, incorporating MTO, Cu^2^⁺, and the exosome secretion inhibitor AMI. To confirm the occurrence of cuproptosis, the authors examined the expression of HSP70 and LIAS and monitored key metabolite changes within the TCA cycle were monitored. Their findings revealed that treatment with CS/MTO-Cu@AMI markedly elevated HSP70 expression while significantly downregulating LIAS. Besides, compared with the control group, the CS/MTO-Cu@AMI group demonstrated a 40% increase in pyruvate and α-ketoglutarate levels in the TCA cycle, while succinate levels dropped by 45%. These results provide compelling evidence supporting the efficacy of CS/MTO-Cu@AMI in inducing cuproptosis [54].

## Ferroptosis Crosstalk with Cuproptosis

The homeostasis of metal ions such as iron and copper is vital for sustaining cellular vitality. Current research has shown that ferroptosis-inducing agents (FINs) hold significant promise in cancer therapy, particularly for cancers susceptible to ferroptosis. Cuproptosis another copper levels. Excessive levels of intracellular copper directly interact with lipoyl moieties within the TCA cycle, leading to the aggregation of lipoylation-associated proteins. This disrupts protein homeostasis and ultimately triggers cell death. Consequently, copper ionophores have attracted considerable interest in cancer treatment strategies. Shen Y et al. conducted a comprehensive analysis of whole-genome genomic data from 33 cancer types sourced from The Cancer Genome Atlas (TCGA) [55]. The results showed that the mutation frequencies of most cuproptosis and ferroptosis regulated factors were comparable across the 33 cancer types. Furthermore, their study results indicated a strong correlation between cuprotosis and ferroptosis. Cuprotosis related factors interacted with ferroptosis factors in the GeneMANIA database. Upon transfection of siRNA and shRNA into A549 cells, the results showed that phosphatase and tensin homolog deleted on chromosome ten (PTEN) knockdown upregulated SLC31A1 expression, mitochondrial transcription factor A (TFAM) knockdown increased ATP7A levels, and ATF2 knockdown inhibited lipoyltransferase 1(LIPT1), indicating a strong association between cuproptosis and ferroptosis factors. Besides, copper can induce or enhance cell sensitivity to iron through multiple mechanisms, as copper plays an essential role as a cofactor for iron-metabolizing enzymes. Copper deficiency may lead to iron deficiency, and one important factor is ceruloplasmin, which contains copper and plays a crucial role in promoting iron binding of iron to carrier proteins, transporting iron in the blood, and facilitating the cellular uptake of iron mediated by transferrin receptor. Copper deficiency may also hinder iron absorption of iron by liver mitochondria, thereby reducing hemoglobin synthesis [56]. Moreover, it has been demonstrated that ferroportin, a pivotal regulator of systemic iron homeostasis, is directly influenced by copper in colon cancer cells [57]. Thus, copper ionophores may elevate iron levels in cells and mitochondria, leading to oxidative stress and ferroptosis in various cancer cells [58, 59]. This similarly indicates that copper is intricately linked with iron metabolism and ferroptosis.

The mitochondrial TCA cycle plays a pivotal role in the emergence of cuproptosis, characterized by the unique lipoylation of four proteins within the cycle: Dihydrolipoamide Branched Chain Transacylase E2 (DBT), Glycine Cleavage System Protein H (GCSH), Dihydrolipoamide S-Succinyltransferase (DLST), and DLAT. Among these, the DLAT protein is an essential component, and its lipoylation by FDX1, coupled with the binding of lipoylated proteins and copper, initiates cuproptosis. Furthermore, copper’s cytotoxic effect compromises mitochondrial integrity by aggregating lipoylated proteins and causing the loss of Fe-S cluster proteins, thereby triggering cuproptosis. Notably, copper-related mitochondrial disease involves alterations in mitochondrial respiration and proteins. This process can be mitigated by employing inhibitors of respiratory chain complexes I and III, such as rotenone and antimycin A, along with mitochondrial pyruvate transporter inhibitors like UK5099 [60]. Glutamine catabolism plays a vital role in cysteine deprivation-induced ferroptosis. As an anaerobic pathway within the mitochondrial TCA cycle, it facilitates ferroptosis by amplifying lipid peroxidation [61]. The mitochondrial TCA cycle and the ETC can trigger ferroptosis by fostering mitochondrial membrane potential hyperpolarization and the buildup of lipid peroxides. Accordingly, it can be inferred that the mitochondrial TCA cycle serves as a convergence point for both ferroptosis and cuproptosis. Further elucidation of the regulatory role of the mitochondrial TCA cycle in these processes is essential for unveiling the interactions between these pathways and exploring their potential applications.

One pathway to induce ferroptosis within the iron-dependent cell death mechanism involves inhibiting the cystine-glutamate transporter (System Xc-). Erastin impedes this transporter, thereby obstructing GSH uptake, an essential cofactor for GPXs. Consequently, the activity of GPXs is downregulated, leading to a reduced cellular antioxidant capacity, accumulation of lipid peroxides, and the onset of oxidative cell death [15, 18]. Moreover, GSH serves as an alternative chelator for copper, playing a role in regulating the concentration of intracellular copper ions [37]. On the other hand, GSH, as a substrate for GPX4, decreases LPO. Wang et al.’s research published in 2023 explored the potential interaction between ferroptosis and cuproptosis in liver cancer cells [62], and their findings suggest that ferroptosis inducers can provoke and amplify cuproptosis in liver cancer cells. The concurrent application of ferroptosis and cuproptosis inducers results in pronounced cell mortality. Specifically, the ferroptosis inducers Sorafenib and Erastin enhance protein lipidation by impeding the degradation of FDX1 protein via mitochondrial matrix-associated proteases. They also inhibit the synthesis of intracellular GSH by restricting cysteine uptake, thereby intensifying cuprum-induced cell death, or cuproptosis. Sorafenib and Erastin primarily facilitate ferroptosis by inhibiting the catalytic subunit (SLC7A11) of system Xc-, which is responsible for importing cysteine for intracellular GSH synthesis [63]. GSH, acting as a copper chelator, can mitigate cuproptosis. Thus, GSH may play a pivotal role in mediating the interaction between ferroptosis and cuproptosis. Furthermore, their research demonstrates that downregulating upstream regulatory factors such as FDX1 and LIAS not only diminishes DLAT aggregation caused by Elesclomol-Cu but also reduces the enhancement of cuproptosis by ferroptosis inducers. These inducers can elevate protein lipidation levels by stabilizing the FDX1 protein. Besides, BSO, an inhibitor of GSH synthesis, can induce ferroptosis. Research by P. Tsvetkov et al. also indicated that BSO could trigger cuproptosis. Building upon the above findings, it can be inferred that can simultaneously target both ferroptosis and cuproptosis, rendering it a promising therapeutic candidate.

The p53 gene is an important oncogene, and its mediated cell cycle inhibition, senescence, and apoptosis play an important role in tumor development. Jiang et al. treated p53-silenced H1299 cells with ROS, with no change in cell availability observed. However, after activating the p53 gene followed by ROS treatment, the cell apoptosis rate reached 90%, which indicated that the cellular antioxidant capacity is significantly reduced by the activation of the p53 gene. After adding ferrostatin-1, an inhibitor of ferroptosis, the cell death rate decreased by about 40%, which revealed that p53 could not only cause apoptosis, but also induce cell ferroptosis. Jiang et al. also found that messenger RNA and protein expression of SLC7A11 were significantly reduced after up-regulation of p53 gene expression, thus confirming SLC7A11 as a new target of p53 gene. The inhibition of System Xc-, a heterodimer composed of SLC7A11 and SLC3A2, resulted in compensatory transcriptional up-regulation of SLC7A11 [64]. Thus, p53 can inhibit the uptake of cystine by System Xc- through the downregulation of SLC7A11 expression, resulting in a reduction in cystine-dependent glutathione peroxidase activity, a decrease in cellular antioxidant capacity, and an increase in lipid ROS, ultimately inducing ferroptosis. Besides, p53 can promote the conversion of pyruvate to acetyl-CoA by facilitating dephosphorylation, thereby activating the PDH complex [65], which is a key component of the TCA cycle required for cuproptosis [37]. Meanwhile, p53 increases acetyl coenzyme A production by enhancing fatty acid oxidation [66, 67] and inhibiting lipid synthesis [68], there by promoting the initiation of the TCA cycle. As a vital nutrient source in the TCA cycle, glutamine is converted to glutamate and subsequently to α-ketoglutarate. Importantly, *p*53 enhances the conversion of glutamine to glutamate by transcriptionally activating glutaminase 2(GLS2) [69, 70]. Interestingly, during glutamine starvation, p53 activated the expression of SLC1A3, which encodes an aspartate/glutamate transporter, to maintain aspartate metabolism and thereby promote the TCA cycle [71]. These findings collectively suggest that *p*53 may enhance sensitivity to cuproptosis by promoting the TCA cycle.

**Fig. 3:**
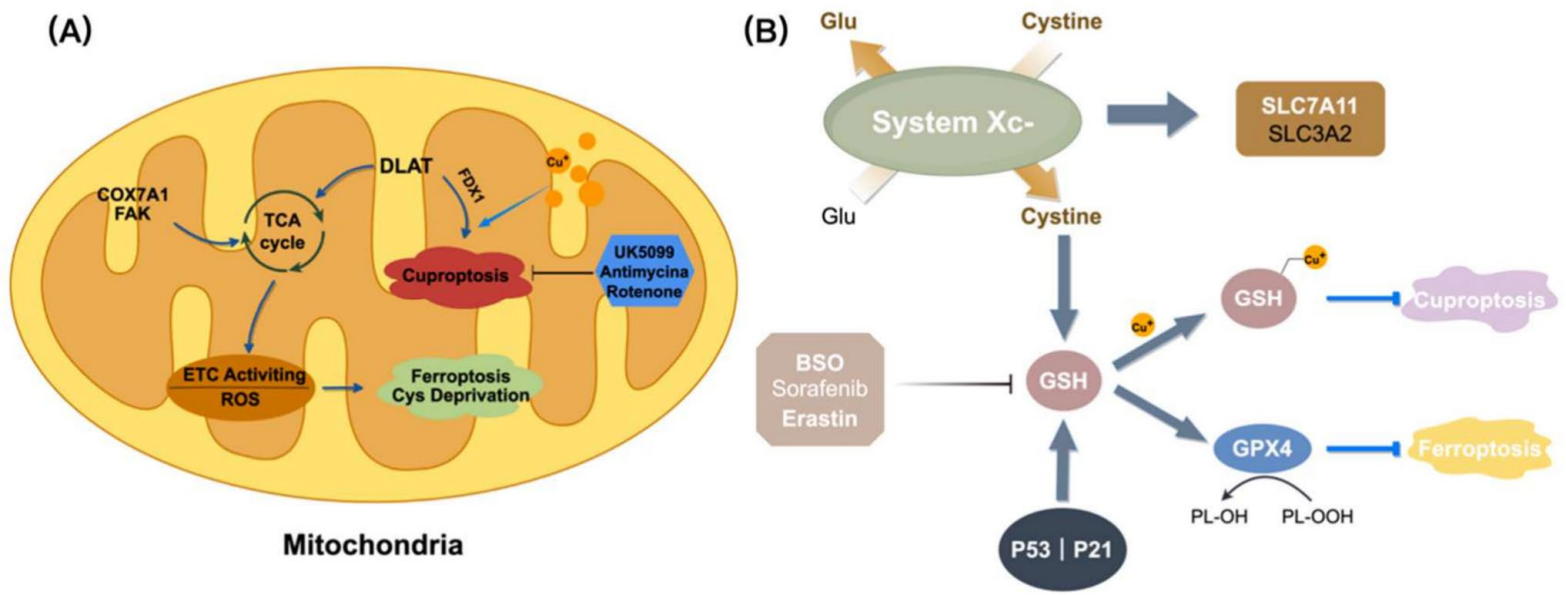
Schematic diagram of interaction between ferroptosis and cuproptosis

The above research suggest a complex interplay between cuproptosis and ferroptosis. The mitochondrial TCA cycle serves as a co-regulatory factor for the depletion of cysteine in both ferroptosis and cuproptosis. Cytochrome c oxidase subunit 7A1 (COX7A1) and focal adhesion kinase (FAK) can accelerate the mitochondrial TCA cycle, increase yield, and promote the depletion of cysteine in both ferroptosis and cuproptosis. Cuproptosis relies on mitochondrial respiration, and mitochondrial respiratory inhibitors (such as rotenone, antimycin, and UK5099) can inhibit cuproptosis (Fig. 3A). GSH is present in nearly every cell and participates in the body’s regulation. SLC7A11 transports cysteine into cells for GSH synthesis. GSH is a powerful reducing agent and acts as a cofactor for GPX4, which degrades lipid peroxides, inhibiting LPO and iron leap. Furthermore, GSH acts as a copper chelator, reducing intracellular copper accumulation and inhibiting cuproptosis. Sorafenib, Erastin, and BSO trigger ferroptosis and cuproptosis by inhibiting GSH synthesis (Fig. 3B). Current evidence suggests that the interaction between cuproptosis and ferroptosis may exacerbate cardiovascular disease progression [72]. Hyperglycemia induces the accumulation of copper and iron in cardiomyocytes and activates signaling pathways related to cuproptosis and ferroptosis, which in turn increases the risk of cardiomyocyte injury and death and promotes the development of cardiovascular complications of diabetes [73]. In addition, cuproptosis and ferroptosis can synergistically promote vascular endothelial cell injury, smooth muscle cell migration and proliferation, and macrophage foam transformation, accelerating the formation and development of atherosclerotic plaques and increasing plaque instability, thus leading to the occurrence of cardiovascular events [73]. Iron-sulfur cluster proteins, such as FDX1, are involved in iron metabolism as well as associated with cuproptosis. The occurrence of cuproptosis leads to a decrease in iron-sulfur cluster proteins, which in turn affects iron metabolism and utilization and promotes ferroptosis. Upon the action of cuproptosis inducers, the expression of mitochondrial Fe-S cluster proteins such as FDX1 decreases significantly, resulting in dysregulated intracellular distribution and metabolism of iron, increased cytosolic iron uptake and iron accumulation in the mitochondria, disrupting the assembly of iron-sulfur clusters and the maintenance of normal cytoplasmic and mitochondrial iron homeostasis, and ultimately leading to ferroptosis [74]. Ferroptosis in arterial dissection (AD) may accelerate the onset and progression of AD by destroying vascular smooth muscle cells (VSMCs) and endothelial cells (ECs) and by weakening vessel wall integrity [75]. In this respect, the iron and malondialdehyde (MDA) levels were significantly elevated in the aortas of AD patients compared with healthy controls, suggesting the presence of excess ferrous iron and lipid hyperoxidative accumulation. Animal studies also suggest that ferroptosis may be involved in the pathogenesis and progression of AD, which provides a theoretical basis and potential targets for the development of new therapeutic strategies. Indeed, both cuproptosis and ferroptosis are widely thought to be closely related to oxidative stress. Impaired mitochondrial function during cuproptosis leads to increased ROS clusters, whereas iron metabolism disorders during ferroptosis also generate large amounts of ROS, and the interaction between the two further exacerbates intracellular oxidative stress levels, leading to cellular damage and death, and thus playing a synergistic role in aortic coarctation development [74].

The mechanisms of metal-mediated cell death pathways are of crucial importance in the future and are expected to provide a fresh perspective on the development of therapeutic drug combinations targeting different cell death modes. In recent years, the discovery of interactions between cuproptosis and ferroptosis has led to new potential targets for the treatment of cardiovascular diseases. Studies have shown that by regulating copper and iron metabolism, inhibiting signaling pathways associated with cuproptosis and ferroptosis, or enhancing the antioxidant capacity of cells, it may play an important role in attenuating the pathophysiological process of cardiovascular diseases as well as improving the prognosis of patients. Table 1 summarizes key features of ferroptosis and cuproptosis, highlighting their cell death types, triggers, molecular mechanisms, proteins, morphological changes, disease associations, and crosstalk with other cell death forms.

**Table 1:** Comparison of ferroptosis and cuproptosis characteristics

Character	Ferroptosis	Cuproptosis
Characterized cell death types	Iron-dependent regulatory cell death	Copper-induced regulatory cell death
Key triggers	Disruption of intracellular iron homeostasis, leading to lipid ROS accumulation	Accumulation of intracellular copper, leading to lipoylation modification of mitochondrial proteins and destabilization of Fe-S cluster proteins
Main molecular mechanisms	1. Lipid peroxidation: Iron promotes the formation of lipid free radicals, leading to lipid peroxidation 2. Inactivation of glutathione peroxidase 4 (GPX4): GPX4 is a key enzyme in the inhibition of ferroptosis, and its inactivation leads to the accumulation of lipid ROS 3. Iron metabolism in cytoplasmic lysate: abnormal regulation of iron transport and storage proteins (e.g. transferrin receptor, ferritin)	1. Mitochondrial dysfunction: accumulation of copper leads to lipoylated modifications of mitochondrial proteins, affecting mitochondrial function 2. Destabilization of Fe-S cluster proteins: copper interferes with the synthesis of Fe-S clusters, affecting the function of key metabolic enzymes 3. Pyruvate dehydrogenase complex (PDH) inhibition: copper inhibits the activity of the PDH complex by interacting with dithiothreitol (DLAT)
Key proteins/genes	GPX4, SLC7A11, ACSL4, ALOXs, FSP1, DHODH, etc	FDX1, DLAT, DBT, GCSH, SLC31A1, LIPT1, DLD, etc
Changes in cellular morphology	1. Mitochondrial decrease in size 2. Rupture of the outer mitochondrial membrane 3. Mitochondrial cristae are reduced or absent 4. Normal-sized nuclei without chromatin condensation	1. Atrophy of mitochondria 2. Rupture of the cell membrane 3. Endoplasmic reticulum and chromatin damage
Association with Disease	Neurodegenerative diseases (e.g. Alzheimer’s disease, Parkinson’s disease), ischemia/reperfusion injury, cancer, etc	Neurodegenerative diseases (e.g. Wilson’s disease), cancer (e.g. hepatocellular carcinoma, head and neck squamous carcinoma), etc
Crosstalk with other forms of cell death	Crosstalk exists with apoptosis, necrosis, and pyroptosis. For example, p53 plays an important role in both ferroptosis and apoptosis	Crosstalk exists with ferroptosis, apoptosis, pyroptosis, etc. For example, in hepatocellular carcinoma, accumulation of copper ions leads to disruption of mitochondrial homeostasis, increases the free iron pool, and induces ferroptosis and cuproptosis

Currently, the development of drugs targeting cuproptosis and ferroptosis, including copper chelators, iron chelators, and antioxidants, is steadily progressing. These drugs are in the development and testing stages, and preliminary results suggest that they may hold promise for the treatment of cardiovascular disease and provide more effective therapeutic options for patients.

## Ferroptosis, cuproptosis, and cardiovascular diseases

The cardiovascular system, an intricate and vital component of the human body, comprises the heart and blood vessels. Through the circulation of blood, it fulfills two primary functions: delivering essential nutrients and oxygen to organs, tissues, and cells to satisfy their physiological demands, and transporting metabolic byproducts, such as carbon dioxide and urea, to the lungs, kidneys, and skin for excretion. Furthermore, the vascular system serves as a critical defense mechanism, as certain blood cells and antibodies can engulf, attack, and neutralize invading bacteria and viruses, as well as the toxins they produce. In recent years, the roles of ferroptosis and cuproptosis in the pathogenesis of various diseases have gained increasing recognition. Modulating these processes, either through inhibition or activation, has emerged as a research focal point for mitigating disease progression. Ferroptosis, in particular, is regarded as a pivotal factor in the onset of various CVDs [76, 77]. The role of cuproptosis as a mode of cell death mediated by copper is also being extensively studied in the context of various diseases. In this section, we focus on summarizing recent advancements in ferroptosis and the effects of cuproptosis on CVDs: Hypertension, Atherosclerosis, HF, and diabetic cardiomyopathy (DCM) and so on.

### Hypertension

Hypertension, as a major risk factor for CVD, triggers changes in arterial structure and function, which in turn lead to an increase in peripheral vascular resistance [78], and pathological cardiac remodeling mediated by hypertension significantly elevates the risk of heart failure. In this pathological process, due to long-term exposure to high pressure loads, results in high Angiotensin II (Ang II) levels. Accordingly, catecholamines and Ang II within the body will continue to stimulate cardiomyocytes, prompting sustained hypertrophy, and triggering interstitial fibrosis, which ultimately results in left ventricular hypertrophy and dilatation. Left ventricular hypertrophy leads to a decrease in coronary blood flow reserve capacity. This limitation becomes pronounced when the body’s consumption of oxygen increases, this blood flow reserve problem is more prominent, which in turn leads to subendocardial ischemia.

A study by Zhang Z et al. found that iron levels in cardiac tissue were significantly upregulated in a hypertensive mouse model infused with Ang II. At the same time, GPX4 and Nrf2 levels were decreased and MDA levels were increased [12]. The peptide hormone Elabela (ELA) is an endogenous ligand for the Apelin receptor, and the ELA-Apelin receptor axis can effectively inhibit heart failure induced by stress load by inhibiting angiotensin-converting enzyme expression as well as Ang II signaling. Treatment with ELA-32 or Fer-1, a ferroptosis inhibitor, improves cardiac function and attenuates myocardial hypertrophy and pathological remodeling by inhibiting ferroptosis. Specifically, ELA was able to antagonize Ang II-mediated ferroptosis in cardiac microvascular endothelial cells (CMVECs) by regulating the IL-6/STAT3 GPX4 signaling pathway and inhibiting the onset of myocardial remodeling and dysfunction [79]. Based on these findings, using the ELA—apj axis as a therapeutic target is expected to provide a novel strategy for the treating hypertensive heart disease. Overall, ferroptosis plays a key role in myocardial remodeling in hypertension, which provides a potential target for the prevention and treatment of hypertension.

Copper, a key component of lysine oxidase and superoxide dismutase (SOD), plays an important role in maintaining arterial collagen and elastin homeostasis [80–84]. Research has shown significant heterogeneity in the correlation between serum copper levels and blood pressure. In this respect, cross-sectional studies have shown elevated serum copper levels in hypertensive patients and rodent models and a positive correlation with disease severity. Studies in European populations have found a positive correlation between diastolic blood pressure and circulating copper levels in children. Besides, studies in Chinese populations have revealed a U-shape correlation between dietary copper intake and hypertension, with an inflection point of 1.57 mg/day. Of note, low serum copper in early pregnancy may increase the risk of gestational hypertension, but this trend was influenced by high pre-pregnancy BMI [85]. Indeed, the lack of consistency in published results may stem from population characteristics (e.g. geography, age, gender) and study design differences. Mechanistic studies have shown that copper is involved in blood pressure regulation through multiple pathways: (1) inhibition of angiotensin-converting enzyme (ACE) activity, and copper deficiency may trigger hypertension by elevating catecholamine or angiotensin levels [86]; (2) copper transporter proteins ATOX1 and ATP7A mediate Ang II regulation of SOD3 [87]; (3) zinc-copper metabolism antagonism may, by altering Zn/Cu ratio affecting vascular function. Recent studies have identified copper metabolism-related genes (*DDIT3, NFKBIA,* etc.) as having potential diagnostic value in pulmonary hypertension [88]. Chen, YF et al. explored the role of Sirtuin 7 (SIRT7) in hypertension-associated myocardial remodeling and cardiac dysfunction and its molecular mechanism by in vitro cellular experiments and in vivo animal experiments. It was found that cuproptosis was significantly activated and negatively correlated with SIRT7 expression in the hearts of hypertensive patients and animal models, and that cuproptosis, myocardial remodeling, and cardiac dysfunction were aggravated upon knockdown of SIRT7, while their overexpression attenuated these symptoms. His results suggest that SIRT7 inhibits cuproptosis and attenuates hypertension-induced myocardial remodeling and cardiac dysfunction by regulating the YAP/ATP7A signaling pathway, providing a new target for the treating of hypertensive heart disease [88].

However, while current studies suggest an association between ferroptosis, copper metabolism disorders and hypertension are associated with each other, the direction of confounding factors and causality has not yet been clarified, and there is a relative lack of basic research, Accordingly,, further in-depth exploration of the underlying mechanisms is needed to alleviate Ang II-induced myocardial injury by inhibiting the metabolic pathway of ferroptosis. Future prospective studies with large samples and in-depth exploration of the copper homeostatic regulatory network as a novel therapeutic target are warranted.

### Atherosclerosis

Atherosclerosis, one of the leading causes of CVD, is essentially a chronic inflammatory disease. Within atherosclerotic lesions, immunoreceptor cells mainly secrete pro-inflammatory cytokines, such as TNF-α, IL-1β, and IL-6, which promote inflammatory responses and exacerbate lesion development [89]. Atherosclerosis is the common pathological basis of myocardial infarction (MI) and Stroke, with plaque rupture and thrombosis being key mechanisms that trigger these two serious cardiovascular events. Upon plaque rupture, its contents (e.g. lipids, necrotic tissue) are exposed to the bloodstream, activating the coagulation system and leading to platelet aggregation and thrombus formation. Complete blockage of a coronary artery by a thrombus will impede blood supply to the myocardium, thus triggering myocardial infarction [90]. Similarly, rupture of an atherosclerotic plaque in the brain can lead to the formation of a blood clot, which may obstruct cerebral circulation, resulting in a cerebral infarction (ischemic stroke). In addition, fragments of atherosclerotic plaque may dislodge and travel with the blood flow into the small arteries of the brain, forming embolisms that can lead to stroke [91].

Atherosclerosis, myocardial infarction and stroke share many common risk factors, including hypercholesterolemia, high blood pressure, smoking, obesity, and genetic factors [92]. Oxidative stress constitutes a key factor in the development of atherosclerosis. Ferroptosis exacerbates cellular damage through lipid peroxidation and may promote the formation and progression of atherosclerotic plaques. In addition, ROS generated during ferroptosis can further damage vascular endothelial cells and foam cells, triggering inflammatory responses and lipid deposition [93]. In patients with hypertensive heart disease, coronary atherosclerotic heart disease, as well as microangiopathy, often complicate the disease. Chronic inflammation and oxidative stress can induce endothelial dysfunction through excess ROS, and endothelial dysfunction not only triggers an inflammatory response and nitric oxide inhibition, but also the accumulation of lipid peroxides associated with ferroptosis, which promotes the onset and progression of cardiovascular and cerebral vascular diseases such as atherosclerosis [94]. Park et al. showed that GPX4 protein levels were significantly downregulated in the early and middle stages of myocardial infarction. When GSH levels were reduced by cysteine deprivation, inhibition of GPX4 sensitized neonatal rat ventricular myocytes to ferroptosis [95]. Both Nrf2 and epithelial mesenchymal transition (EMT) have been associated with myocardial infarction. Current evidence suggests that Nrf2, a major transcription factor regulating the antioxidant response of various types of cells, inhibits ferroptosis by protecting cells from lethal ROS stress [96]. In myocardial infarction, EMT signaling is activated, leading to ferroptosis within cardiomyocytes. Therefore, reducing residual iron within the myocardium may be a potential therapeutic target for reducing adverse ventricular remodeling in patients with reperfused myocardial infarction.

Accumulation of copper ions is closely associated with the onset and progression of atherosclerosis. Epidemiological studies have shown that high serum copper levels are associated with an increased risk of atherosclerotic disease. In atherosclerotic plaques, elevated copper levels may promote plaque destabilization through the induction of oxidative stress and inflammatory responses. Cuproptosis leads to impaired cellular energy metabolism and cell death by interfering with mitochondrial function and the TCA cycle [97]. In addition, copper ions activate the NF-κB signaling pathway and promote inflammatory responses, which further exacerbate the progression of atherosclerosis. He et al. identified differential expression of cuproptosis-related genes (A-CRGs) between the atherosclerosis and control groups and analyzed their functions. Five A-CRGs were screened, which were mainly associated with copper metabolism and immune-inflammatory response, yielding an atherosclerosis diagnostic model with good diagnostic value. It was found that cuproptosis-related genes such as FDX1, SLC31A1, and GLS played a key role in atherosclerosis, with FDX1 and SLC31A1 up-regulated in atherosclerotic plaques, while GLS was down-regulated; GLS was expressed mainly in vascular smooth muscle cells, while SLC31A1 was mainly localized in macrophages of atherosclerotic lesions, which are potential diagnostic markers of atherosclerosis. This study also suggested that cuproptosis could be a potential pathogenic mechanism of atherosclerosis, and the related genes are expected to become diagnostic markers and molecular subtype recognition markers of atherosclerosis, which providing novel insights for the study of the pathogenesis of atherosclerosis, as well as the exploration of new diagnostic markers, and the exploration of personalized therapeutic approaches [98].

In addition, Yang, M et al. integrated three myocardial infarction-related bulk datasets from the GEO database, and compared 6378 differentially expressed genes (DEGs) with 49 cuproptosis-related genes. They identified 16 cuproptosis-related genes and screened four key genes, namely Dbt, DLAT, Ube2d1, and Ube2d3. Focusing on the Ube2d3 gene, a mouse model of myocardial infarction was constructed, and Ube2d3 expression and the level of cuproptosis-related proteins were detected by qRT-PCR and Western Blot to elucidate the correlation between cuproptosis and the development of myocardial infarction. TTC staining of the myocardial infarction mouse model showed obvious white infarcted areas in the myocardial tissue, and HE staining showed necrosis, edema, neutrophil infiltration and disordered arrangement of myocardial cells. Immunofluorescence also showed significant neutrophil infiltration in the myocardial tissue, and the inflammatory factors (IL-6, IL-1β, TNF-α, and MCP-1) were elevated, which indicated that the myocardial tissue was in a state of immunoactivation. Ube2d3 expression was significantly up-regulated in the myocardial tissues of mice in the myocardial infarction group, and the lipid acylation levels of cuproptosis-associated proteins FDX1 and SLC31A1, as well as DLAT, were significantly increased, while significant aggregation of DLAT was observed. Overall, this study suggested that Ube2d3 could influence the progression of myocardial infarction by regulating cuproptosis and contribute to myocardial infarction, which is expected to be a potential target for myocardial infarction therapy [99].

Both ferroptosis and cuproptosis involve oxidative stress mechanisms, and oxidative stress plays a key role in the development and progression of atherosclerosis [93]. Excessive accumulation of both copper and iron ions induces the generation of ROS, which damages cell membranes and DNA, thereby promoting the development of atherosclerosis [100]. In addition, copper ions can influence cellular sensitivity to ferroptosis by regulating the expression of genes critical for ferroptosis (e.g. GPX4). It has been shown that copper chelators can specifically reduce cellular sensitivity to ferroptosis without affecting other forms of cell death [93]. This interaction between copper ions and ferroptosis may play an essential role in the pathology of atherosclerosis, especially during plaque destabilization and rupture.

In summary, ferroptosis and cuproptosis are closely related to atherosclerosis through oxidative stress and cell death mechanisms. Further study of the interactions between these cell death pathways is vital for the development of novel therapeutic strategies against atherosclerosis.

### Heart failure

Heart failure stands as the predominant cause of mortality in CVDs. A critical pathogenic element in the progression of lethal HF is the loss of terminally differentiated cardiomyocytes. However, the precise mechanism underlying cardiomyocyte death remains elusive. Ferroptosis, an iron-dependent, non-apoptotic form of cell death, can be prevented or even reversed through iron chelation [101, 102]. Fang et al. elucidated the significant role of iron dysregulation in the onset and progression of CVDs, along with its regulatory mechanisms [76]. In this study, various cell death inhibitors were used to establish animal models associated with cell death pathways. The study found that apoptosis-specific iron inhibitors could significantly reduce cardiac damage caused by doxorubicin or ischemia–reperfusion surgery. To identify key regulators of the ferroptosis in Adriamycin cardiomyopathy models, transcriptomic sequencing (RNA-seq) revealed significant upregulation of heme oxygenase-1 (Hmox1). Hmox1 is widely recognized as a powerful cardioprotective protein [103, 104]. Activation of Hmox1 reportedly facilitates the release of free iron ions from heme, leading to their accumulation in cardiomyocytes and subsequently inducing ferroptosis. Further investigations revealed that iron overload and ferroptosis predominantly occur in the mitochondria, rather than in the cytoplasm of cardiomyocytes, following adriamycin exposure.

Besides, in a separate study, experiments using conditional knockout Ferritin H (Fth) allele mice revealed a separate study, discovering a significant increase in ferroptosis within their cardiomyocytes, accompanied by elevated ROS levels and an enhanced susceptibility to HF. To explore the molecular regulatory mechanisms, heart samples that underwent RNA-seq screening revealed that ferroptosis and its associated GSH metabolic pathway were markedly upregulated in Fth knockout myocardial tissues. Further studies uncovered that a deficiency in intracellular cystine and reduced GSH, due to the downregulation of SLC7A11*,* a functional subunit of the cystine/glutamate antiporter on the cardiomyocyte membrane is the primary mechanism inducing ferroptosis in cardiomyocytes. By constructing Fth-deficient mice with SLC7A11 overexpression, Fang X et al. found that this overexpression could enhances intracellular cystine levels, subsequently increasing intracellular GSH and reversing ferroptosis in Fth-deficient cardiomyocytes, along with their associated HF phenotype. These findings not only elucidate GSH deficiency as a molecular mechanism inducing cardiac ferroptosis in Fth knockout mice, but also suggest that SLC7A11 could be a pivotal therapeutic target for treating ferroptosis-related cardiac diseases [105].

Mitochondria are pivotal in cardiac function. As a bioenergetic disorder characterized by profound mitochondrial dysfunction, substantial evidence indicates that both cellular energy production and transfer are compromised in patients with HF [106]. Mitochondrial dysfunction is widespread, irrespective of the underlying etiology of HF in patients. Given that mitochondrial dysregulation is a pivotal contributor to the progression of HF [107, 108], mitochondria have emerged as an important therapeutic target in HF [109, 110].

It is now understood that the primary role of mitochondria is adenosine triphosphate (ATP) generation through electron transport in the ETC complex. AMP-activated protein kinase (AMPK) plays a crucial role in regulating mitochondrial homeostasis [111]. Ferroptosis can result in cardiac mitochondrial damage through iron overload, including mitochondrial structural abnormalities, altered mitochondrial membrane potential, and increased mitochondrial ROS, which are also considered essential features of ferroptosis [112]. Research has demonstrated that during energy depletion, the energy sensor AMPK becomes activated, consequently inhibiting ferroptosis [113]. The underlying mechanism is attributed to AMPK activation by the upstream kinase liver kinase B1(LKB1), which hinders cellular lipid synthesis by inhibiting the phosphorylation of acetyl-CoA carboxylase 1 (ACC1) and other potential substrates essential for lipid biosynthesis [114].

Furthermore, mitochondria represent a primary target of cuproptosis, characterized by oxidative damage to mitochondrial membranes and compromised enzymatic function within the TCA cycle [115, 116]. Copper primarily affects the TCA cycle rather than directly targeting the ETC. This is evidenced by the observation that while basal respiration remains largely unaffected, the reserve capacity is diminished. Moreover, protein lipoylation is essential for copper interaction, as copper binds to lipoylated TCA cycle proteins, leading to the disruption of Fe-S cluster proteins, inducing proteotoxic stress and amplifying toxicity. Given that copper toxicity arises from copper overload and is closely linked to lipoylated TCA cycle proteins, it is plausible to consider it a contributor to HF and a potential therapeutic avenue. The deletion of FDX1 has been shown to confer cellular resistance to cuproptosis. Besides, FDX1 is highly correlated with the expression of lipoylated proteins, and its knockdown results in a complete loss of protein lipoylation [37]. Therefore, we speculate that FDX1 may be a key target for cuproptosis-based treatment of HF, which regulates mitochondrial protein hyperacetylation and reduces cell death in HF. Therefore, the study suggests that FDX1 may be a key target for cuprotoosis-based treatment of HF, given that FDX1 regulates mitochondrial protein hyperacetylation, reducing HF cell death. Copper chelators not only repair rat myocardial mitochondria but also restore proliferator-activated receptor γ coactivator-1α, improving cardiac pumping function [117].

Mitochondria play a central role in both the mechanisms of cell death and the development of HF. Accordingly, the development of drugs targeting mitochondria holds great promise for the correction of ferroptosis and cuproptosis in the treatment of HF. However, no breakthroughs have yet been achieved since the key underlying mechanisms are not well understood.

Several studies have shown gender differences in ferroptosis in heart failure. Regitz-Zagrosek et al. suggested that ferroptosis plays an essential role in the pathogenesis of heart failure and exhibits gender differences. Animal experiments have shown that female rat cardiomyocytes respond differently to ischemic stress than males, which may be related to the protective effect of estrogen on the myocardium [118]. López-Vilella et al. have also shown that females exhibit a different prognosis in cardiac disease, which may be related to sex differences in cardiac iron metabolism. Studies on patients with heart failure revealed that women usually have lower levels of cardiac iron overload than men, and cardiac iron overload is an important trigger of ferroptosis, accounting for the relatively better prognosis of female heart failure patients. Meanwhile, while it has been established that heart failure is associated with abnormal copper metabolism, the gender differences in cardiac copper metabolism and the mechanism of its effect on cuproptosis are unclear, warranting further study [118]. In addition, differences in cardiac iron metabolism between men and women in heart failure patients have been reported, with male heart failure patients often experiencing more severe cardiac iron overload, which increases the risk of ferroptosis and leads to a relatively higher mortality rate in male heart failure patients [119]. Although few studies have been conducted on cardiac cuproptosis, it has been shown that copper plays an essential role in heart disease and that there may be gender differences. For example, a study found that men and women with myocardial infarction exhibited different levels of cardiac copper [120], which may influence the occurrence of cuproptosis, although the exact mechanism underlying the gender difference is unclear and requires further in-depth study.

### Diabetic cardiomyopathy

Cardiovascular diseases remain a predominant cause of mortality among patients with diabetes, with CAD and ischemic cardiomyopathy being particularly prevalent. Individuals with diabetes often initially exhibit impaired diastolic cardiac function while maintaining preserved systolic function, a condition known as heart failure with preserved ejection fraction (HFpEF) [121]. Various mechanisms contribute to the deterioration of cardiac function in diabetes. These encompass the heart’s exposure to a diabetic milieu marked by elevated blood glucose, along with increased levels of fatty acids (FA) and cytokines. High glucose levels enhance the enzymatic O-GlcNAcylation of myocardial proteins, while the augmented formation of advanced glycation end-products (AGEs), resulting from non-enzymatic glycosylation, further exacerbates detrimental effects [122]. Besides, there is a close connection between diabetic complications and ferroptosis. Hyperglycemia leads to excessive production of ROS and induces oxidative stress in various organs of diabetic patients. High glucose can cause iron overload, and iron imbalance can induce ROS production and promote oxidative stress, ultimately leading to ferroptosis [123]. An earlier study by Kazi TG revealed elevated mean levels of Cu and Fe in the scalp hair and blood of patients compared to non-diabetic individuals. This finding indirectly indicates that diabetic patients may experience increased concentrations of Fe and Cu in the body and potentially in the heart [124].

AGEs are reportedly key pathogenic factors that contribute to the development of DCM, primarily by elevating oxidative stress levels. A study by Wang X et al. demonstrated that ferroptosis is the primary mechanism of AGE-induced cell death and plays a crucial role in AGE-induced endothelial cell dysfunction [125]. Moreover, in the hearts of DCM mice subjected to a high-fat diet and streptozotocin (STZ) injection, increased expression of the lipid peroxidation marker MDA and the ferroptosis marker Ptgs2 was observed, along with a reduction in ferritin levels, *SLC7A11* expression, GSH levels, and the GSH/GSSG ratio. Consistent with observations in AGE-treated endothelial cells, the level of active iron was significantly elevated in heart homogenates of diabetic mice compared to healthy controls. Moreover, treatment of diabetic mice with a ferroptosis inhibitor, liproxstatin-1, demonstrated that it could mitigate the decline in diastolic function three months post-diabetes onset. These findings collectively suggest that ferroptosis plays a role in the pathogenesis of DCM. A study by S. Du et al. demonstrated that Cagliflozin could attenuate high glucose-induced ferroptosis in cardiomyocytes by downregulating FTN-H expression in DCM mice and cells, while upregulating cystathionine-glutamate reverse transporter protein (xCT). This suggests that cagliflozin inhibits ferroptosis by balancing cardiac iron homeostasis and promoting the Xc/GSH/GPX4 axis in the DCM system [126]. Further experiments indicated that sulforaphane (SFN) could inhibit ferroptosis in DCM cardiomyocytes through activation of Nrf2 mediated by AMP-AMPK [127, 128].

Compromised mitochondrial function leads to the production of substantial quantities of ROS and free iron, which subsequently catalyze LPO [129]. Conversely, mitochondrial dysfunction results in diminished cysteine production and GSH depletion, ultimately impairing the detoxification of lipid peroxides by GPX4 [61, 130]. Mitochondrial repair or mitochondrial-specific overexpression of GPX4 can alleviate LPO [22, 131]. Research by Chen et al. has demonstrated the beneficial effects of isoproterenol (ISO) treatment on cardiac microcirculation in diabetic patients. ISO treatment inhibits the mitochondrial translocation of ACSL4 via the PRDX2-MFN2 pathway, ultimately suppressing ferroptosis and protecting the structure and function of cardiac microvessels. These findings also substantiate that inhibiting mitochondrial-derived iron depletion may be a promising therapeutic strategy for addressing cardiac microvascular damage and mitochondrial dysfunction.

Besides, studies have demonstrated that in individuals and rodents with DCM, circulating copper concentrations are increased, with extracellular myocardial Cu^2^⁺ levels rising two to threefold, while intracellular myocardial copper levels are decreased. This disparity indicates impaired copper uptake by cardiomyocytes, as evidenced by reduced myocardial copper levels alongside elevated systemic and total cardiac copper concentrations [132, 133]. The glycosylation of proteins, resulting in the formation of AGEs, represents a harmful consequence of hyperglycemia associated with diabetes and metabolic syndrome [134]. In the hearts of diabetic rats, elevated extracellular Cu^2^⁺ enhances the expression of genes such as TGF-β, Smad4, and collagen, resulting in collagen deposition and an increased formation of collagen AGEs. These processes contribute to vascular injury and heighten susceptibility to IHD [135]. In DCM, elevated extracellular Cu^2^⁺ is reportedly associated with components of the extracellular matrix, such as collagen [136, 137]. Huo et al. reported that copper could significantly affect cardiomyocyte dysfunction induced by AGEs in DCM. They proposed that AGE-induced cuproptosis could represent a novel mechanism for DCM. This research indicated that an excessive increase in AGEs and copper in diabetic patients could trigger the upregulation of the copper transporter SLC31A1 via the ATF3/SPI1 pathway. This leads to copper accumulation in cardiomyocytes, disrupting copper homeostasis and promoting its proliferation. Besides, a decline in Fe-S cluster proteins and the lipidation of DLAT and DLST could exacerbates mitochondrial dysfunction in cardiomyocytes, resulting in myocardial impairment. Overall, these findings suggest that AGE-CuCl2-induced cuproptosis through the ATF3/SPI1/SLC31A1 pathway may serve as a potential therapeutic target for DCM [53].

In addition to the aforementioned therapeutic potential of ferroptosis and cuproptosis for CVDs, numerous studies have revealed their significant roles as potential targets for treating osteoporosis (OP). Moreover, extensive research has demonstrated a correlation between bone loss and cardiovascular risk, primarily mediated through vascular calcification [138, 139]. Ni et al. showed ferroptosis is involvement in the differentiation process of osteoclasts and found that 2-methoxyestradiol targeting of HIF-1α and ferritin promotes the occurrence of ferroptosis in osteoclasts for the treatment of OP [140]. In Shi, J et al. found that P300 deficiency could promote ferroptosis in vascular smooth muscle cells mediated by activation of the HIF-1α/HMOX1 axis [141]. IMa et al. discovered that elevated glucose levels could trigger ferroptosis in the bone tissue of diabetic rat models by increasing GSH consumption. Through both in vivo and in vitro experiments with varying melatonin concentrations, they found that melatonin could inhibit osteoblast ferroptosis by activating the Nrf2/HO-1 signaling pathway, thereby enhancing the osteogenic capacity of these cells [142]. Furthermore, Yang et al. demonstrated that the deterioration of bone trabeculae and bone loss were markedly mitigated in diabetic osteoporosis (DOP) mice following treatment with the ferroptosis inhibitor Fer-1. GPX4 expression in these diabetic mice was significantly upregulated by Fer-1 treatment, suggesting that Fer-1 restores redox homeostasis and enhances osteoblast resistance to ferroptosis [143]. These findings collectively underscore the pivotal role of oxidative stress and LPO, induced by iron overload, in the pathogenesis of OP. The hypoxic environment within bone tissue and glycolysis provides metabolic pathways that can inhibit cuproptosis, thereby fostering the survival and proliferation of osteoblasts, osteoclasts, effector T cells, all mediating osteoporosis progression. Moreover, research by Li et al. indicated that key genes regulating cuproptosis were linked to the development of OP. These insights suggest that modulating ferroptosis in osteoclasts and osteoblasts, alongside targeting genes associated with cuproptosis, could offer promising strategies and directions for osteoporosis treatment [144]. OP is a significant contributor to morbidity and mortality among the elderly. The association between OP and CVD is attributed to shared risk factors, including age, smoking, alcohol consumption, physical inactivity, and menopause. The potential mechanisms linking osteoporosis with atherosclerosis encompass bone and vascular mineralization, estrogen deficiency, elevated plasma homocysteine levels, lipid oxidation, and chronic inflammatory processes [145, 146]. In osteoporosis, akin to atherosclerosis, lipid oxidation results in deposits beneath the endothelium of the bone vascular system [146]. Notably, calcium deposition and atherosclerosis have been intricately linked to the association between OP and CVDs [147–149]. Ma et al. conducted both in vitro and in vivo experiments demonstrating that metformin (Met) could inhibit ferroptosis by modulating the POSTN/p53/SLC7A11 axis, thereby ameliorating lipid-induced vascular calcification [150]. Consequently, further exploration is warranted to determine whether targeting ferroptosis and cuproptosis could offer promising therapeutic strategies for OP, potentially alleviating or treating CVDs.

In addition, ferroptosis plays an important role in other types of cardiovascular disease along with cuproptosis. Although doxorubicin is a commonly used chemotherapeutic agent treating many cancers, its cardiotoxicity (Doxorubicin-Induced Cardiomyopathy (DIC)) limits clinical application. Ferroptosis and cuproptosis play critical roles in DIC, with ferroptosis triggering cardiomyocyte death through iron metabolism disorders, increased oxidative stress, and mitochondrial dysfunction, and cuproptosis potentially exacerbating myocardial injury through aberrant copper metabolism and disruption of antioxidant defense systems [100, 151]. While therapeutic strategies targeting ferroptosis have yielded favorable protective effects, studies related to cuproptosis are in the exploratory stage, which may provide new targets for cardiotoxicity prevention and treatment of doxorubicin in the future [152].

Aortic stenosis (AS) is a common cardiovascular disease characterized by aortic valve stenosis and blockage of blood ejection from the left ventricle to the aorta. In AS, ferroptosis is widely thought to lead to lipid peroxidation and cell membrane damage through dysregulated iron metabolism, exacerbating oxidative stress, which in turn damages valve cells and exacerbates valve stenosis [153, 154]. Cuproptosis, on the other hand, may damage valve cells by generating hydroxyl radicals through oxidative stress involving copper ions, leading to DNA damage and lipid peroxidation, disrupting lipid metabolism to promote lipid deposition in the valve, and binding copper ions to fatty acylated proteins to trigger protein aggregation and dysfunction [155, 156]. Table 2 summarizes therapeutic approaches for ferroptosis and cuproptosis, detailing the mechanisms of antioxidants, metabolic regulators, gene therapy, and combined treatments.

**Table 2:** Comparison of types of treatment programs for ferroptosis and cuproptosiss

Type of treatment program	Ferroptosis	Cuproptosis
Antioxidants	1. Ferroptosis inhibitors: e.g. Ferrostatin-1, which inhibits the accumulation of lipid ROS 2. GPX4 activators: e.g. RSL3 antagonist, which enhances GPX4 activity 3. Iron chelators: e.g. Deferoxamine, which reduces intracellular iron availability	1. Copper ionophores: e.g. Elesclomol, which brings copper into the cell and induces cuproptosis 2. Copper chelators: e.g. Bis(thiosemicarbazone) ligand, which reduces intracellular copper accumulation 3. Lipoic acid pathway modulators: e.g. Lipoic acid, which affects copper-induced cell death
Antioxidants	1. Glutathione precursors: e.g. N-acetylcysteine (NAC), which enhances glutathione synthesis and reduces lipid ROS 2. Vitamin E: inhibits lipid peroxidation	1. Antioxidants: e.g. NAC can also be used to attenuate ROS damage in cuproptosis 2. Other antioxidants: e.g. Tempol which reduces ROS production
Metabolic modulators	1. Glutamine antagonists: e.g. Elesclomol, which affect metabolic processes associated with ferroptosis 2. Modulators of lipid metabolism: e.g. ACSL4 inhibitors, reduce lipid peroxidation	1. Modulators of mitochondrial function: e.g. electron transport chain complex I/III inhibitors (e.g. Rotenone and Antimycin A), which attenuate copper-induced mitochondrial damage 2. Modulators of the pyruvate dehydrogenase complex: e.g. UK5099, modulates mitochondrial metabolism
Gene therapy	1. GPX4 gene therapy: inhibit ferroptosis by enhancing the expression of GPX4 2. Iron metabolism-related gene editing: editing of genes such as SLC7A11, FSP1, which can regulate the ferroptosis process	1. Gene editing of FDX1, DLAT, etc.: control cuproptosis by regulating the expression of these genes 2. Editing of copper transport-related genes: such as SLC31A1, ATP7B, and other genes, which can regulate intracellular copper homeostasis
Combination therapy	1. Combination of ferroptosis inhibitor and other anticancer drugs: for example, Ferrostatin-1 is combined with chemotherapeutic drugs to enhance the anticancer effect 2. Combination of ferroptosis and apoptosis inhibitors: it can inhibit multiple cell death pathways simultaneously and enhance the therapeutic effect	1. Cuproptosis inducer combined with other anticancer drugs: e.g. Elesclomol combined with chemotherapeutic drugs to enhance the anticancer effect 2. Cuproptosis inducer combined with ferroptosis inhibitors can synergistically induce cell death in some cancers

In conclusion, ferroptosis and cuproptosis play crucial roles in the development of cardiovascular disease. Future studies should further explore the specific details of these mechanisms, and an in-depth study of their mechanisms and interactions will help develop new therapeutic strategies, improve the therapeutic efficacy of cardiovascular diseases and the prognosis of patients, and provide a more comprehensive perspective and potential targets for clinical treatment.

## Conclusion

In recent years, extensive research has illuminated the mechanisms of ferroptosis, whereas cuproptosis, a newly identified form of metal-mediated cell death, remains relatively underexplored. As investigations into cuproptosis advance, new avenues emerge for understanding its role in various diseases. Both ferroptosis and cuproptosis are characterized by the excessive accumulation of metal ions, and recent studies suggest a strong correlation between them, with GSH potentially playing a pivotal role in mediating the interaction between these two forms of cell death. Besides, the p53 gene not only induces cell death via ferroptosis but also enhances cellular sensitivity to cuproptosis by promoting the TCA cycle. Consequently, could the co-regulation of these two forms of cell death through the p53 gene offer a novel therapeutic approach? We eagerly anticipate further research to elucidate the direct connections between these regulatory mechanisms and to reveal the development of compounds that target both pathways concurrently.

Ferroptosis is well-known to play a pivotal role in the pathogenesis of numerous CVDs, suggesting that its inhibition could serve as a promising therapeutic strategy. In contrast to its extensive exploration in oncology, cuproptosis has received comparatively less attention in the cardiovascular realm. Nonetheless, given the dysregulation of both ferroptosis and cuproptosis in cardiovascular conditions, restoring iron and copper homeostasis emerges as a compelling target for treatment. Recent studies have highlighted mitochondria as a central focus of ferroptosis and cuproptosis-induced cell death, making them a critical therapeutic target in HF. Both ferroptosis and cuproptosis detrimentally affect mitochondrial function, exacerbating HF progression. Thus, drug development aimed at mitochondria holds significant potential for HF treatment.

Overall, ferroptosis and cuproptosis, two distinct forms of metal-mediated cell death, are intricately linked. A deeper investigation into the mechanisms governing both iron metabolism and cuproptosis could enable more precise regulation of the onset and progression of CVDs. As research into these pathways continues to evolve, we anticipate significant breakthroughs and advancements that could offer new hope for the treatment and prevention of cardiovascular conditions.

## Data Availability

No datasets were generated or analysed during the current study.

## Abbreviations

ACC1: Acetyl CoA carboxylase 1
ACO2: Aconitase 2
ACSL 4: Acyl-CoA synthetase long chain family member 4
AD: Aortic dissection
AGEs: Advanced glycation end-products
AMPK: AMP-activated protein kinase
Ang II: Angiotensin II
AS: Aortic stenosis
ATP: Adenosine triphosphate
ATP7A: ATPase A
ATP7B: ATPase copper transporting Beta
BSO: Butylthionine sulfoimide
CADs: Coronary artery diseases
COX7A1: Cytochrome c oxidase subunit 7A1
CTR1: Copper transport protein 1
CMVECs: Cardiac microvascular endothelial cells CVDs, cardiovascular diseases
DBT: Dihydrolipoamide branched chain transacylase E2
DCM: Diabetic cardiomyopathy
DEGs: Differentially expressed genes
DIC: Doxorubicin-induced cardiomyopathy
DLAT: Dihydrolipoamide S-acetyltransferase
DLST: Dihydrolipoamide S-Succinyltransferase
DMT1: Divalent metal transporter 1
ECs: Endothelial cells
EMT: Mesenchymal transition
ELA: Elabela
ETC: Electron transport chain
ETFDH: Electron transfer flavoprotein dehydrogenase
FA: Fatty acids
FAK: Focal adhesion kinase
FDX1: Ferredoxin 1
Fe-S: Iron-sulfur
Fe-S clusters: Iron-sulfur cluster
FINs: Ferroptosis-inducing agents
Fth: Ferritin H
FTH: Ferritin heavy chain
FTL: Ferritin light chain
GCSH: Glycine cleavage system protein H
GLS2: Glutaminase 2
GPX4: Glutathione peroxidase 4
GSH: Glutathione
GSSG: Oxidized glutathione
HF: Heart failure
HFpEF: Heart failure with preserved ejection fraction
Hmox1: Heme oxygenase-1
HSP70: Heat shock protein 70
IHD: Ischemic heart disease
ISO: Isoproterenol
LIAS: Lipoic acid synthase
LIP: Labile iron pool
LIPT1: Lipoyltransferase 1
LKB1: Liver kinase B1
LPCAT3: Lysophosphatidylcholine acyltransferase3
LPO: Lipid peroxidation
MDA: Malondialdehyde
MI: Myocardial infarction
NCOA4/FTH1: Nuclear receptor coactivator 4/ferritin heavy chain1
NDUFS8: NADH: ubiquinone oxidoreductase core subunit S8
NDUFV1: NADH: ubiquinone oxidoreductase core subunit V1
Nrf2: Nuclear factor-erythroid 2 related factor 2
OP: Osteoporosis
PDC: Pyruvate dehydrogenase complex
PDH: Pyruvate dehydrogenase
PUFA-CoA: Polyunsaturated fatty acid coenzyme A
PUFA: Polyunsaturated fatty acids
PUFA-pls: Polyunsaturated-fatty-acid–containing phospholipids
RCD: Regulated cell death
ROS: Reactive oxygen species
SFN: Sulforaphane
SIRT7: Sirtuin 7
SLC3A2: Solute carrier family 3 member 2
SLC7A11: Solute carrier family 7 member 11
SLC31A1: Solute carrier family 31 member 1
STEAP3: Six-transmembrane epithelial antigen of prostate 3
STZ: Streptozotocin
TCGA: The Cancer Genome Atlas
TCA cycle: Tricarboxylic acid cycle
TFAM: Mitochondrial transcription factor A
TF: Transferrin
TFR1: Transferrin receptor 1
VDAC2/3: Voltage-dependent anion channel2
VSMCs: Vascular smooth muscle cells
4-HNE: 4-Hydroxynonenal
